# A large-scale solar dynamics observatory image dataset for computer vision applications

**DOI:** 10.1038/sdata.2017.96

**Published:** 2017-07-25

**Authors:** Ahmet Kucuk, Juan M. Banda, Rafal A. Angryk

**Affiliations:** 1 Department of Computer Science, Georgia State University Atlanta 30302-3987, USA

**Keywords:** Solar physics, Computer science

## Abstract

The National Aeronautics Space Agency (NASA) Solar Dynamics Observatory (SDO) mission has given us unprecedented insight into the Sun’s activity. By capturing approximately 70,000 images a day, this mission has created one of the richest and biggest repositories of solar image data available to mankind. With such massive amounts of information, researchers have been able to produce great advances in detecting solar events. In this resource, we compile SDO solar data into a single repository in order to provide the computer vision community with a standardized and curated large-scale dataset of several hundred thousand solar events found on high resolution solar images. This publicly available resource, along with the generation source code, will accelerate computer vision research on NASA’s solar image data by reducing the amount of time spent performing data acquisition and curation from the multiple sources we have compiled. By improving the quality of the data with thorough curation, we anticipate a wider adoption and interest from the computer vision to the solar physics community.

## Background & Summary

NASA’s Living With a Star (LWS) program initiated the Solar Dynamic Observatory (SDO) mission on February 11, 2010. The SDO mission is an attempt to obtain scientific knowledge that can help to understand the influence of the Sun on Earth^1^. SDO is a spacecraft in an inclined geosynchronous orbit around Earth capturing full-disk images of the Sun for up to ten years. As part of the SDO mission, three independent instruments are designed for different purposes^2^. The Atmospheric Imaging Assembly (AIA) is built in partnership with Lockheed Martin Solar and Astrophysics Laboratory (LMSAL). Every ten to twelve seconds, the AIA module captures high-definition (4096×4096) full-disk images of the Sun in eight different wavelengths using four AIA telescopes^3^. Our dataset is built from the images produced by the AIA module. There are two other modules, namely the Extreme Ultraviolet Variability Experiment (EVE) and Helioseismic and Magnetic Imager (HMI), which generate a different type of imaging beyond the scope of this dataset.

Because of the large number of images taken by the SDO mission, automatically detecting features in the images is a necessary part of the mission. Several different international groups, such as the SDO Feature Finding Team (FFT), developed modules to detect features on the full-disk images^2^. The SDO FFT modules report detected events to the Heliophysics Event Knowledge Base (HEK) system which provides access to the data through a public Application Programming Interface (API)^4^. However, event records in the HEK are not limited to SDO FFT reports. The HEK receives event reports from several different institutes such as the National Oceanic and Atmospheric Association (NOAA), Jet Propulsion Laboratory (JPL) Stanford, Smithsonian Astrophysical Observatory (SAO), Royal Observatory of Belgium (ROB), and LMSAL. The HEK API can be used to retrieve solar event records reported from these institutes. As a major shortcoming, the HEK repository does not deliver image data. In order to retrieve image data, we used Helioviewer, which is funded by NASA and provides a public API for accessing high definition (4096×4906) full-disk images of the Sun in compressed JPEG 2000 format^5^.

Since the purpose of the SDO mission is broad in nature, different research domains use SDO data. HEK and Helioviewer-like systems are developed for providing generic data for various research purposes. Since the data collection requires a significant amount of time and attention to detail, researchers need to cautiously collect and prepare appropriate data from these sources to avoid form errors during the preparation process. To overcome these issues, we are presenting a ready-to-use dataset for event retrieval applications. To the best of our knowledge, this work is the first SDO 4 K resolution image dataset prepared for image retrieval applications.

The majority of our dataset is prepared for the research area of Content-Based Image Retrieval (CBIR). For this purpose, we include the full-disk image of the Sun with temporal and spatial features of the event records in the dataset. Additionally, we provide ten different image parameters that are extracted from these full-disk images. As determined in our previous research^6^, these ten different image parameters were selected as the best for the solar domain. As a result of several tests to validate and clean the dataset, we make available approximately 260,000 images taken by the AIA module with image parameters and 270,000 event records in a well-prepared format for future research.

## Methods

### Event records from HEK

**Figure 1:**
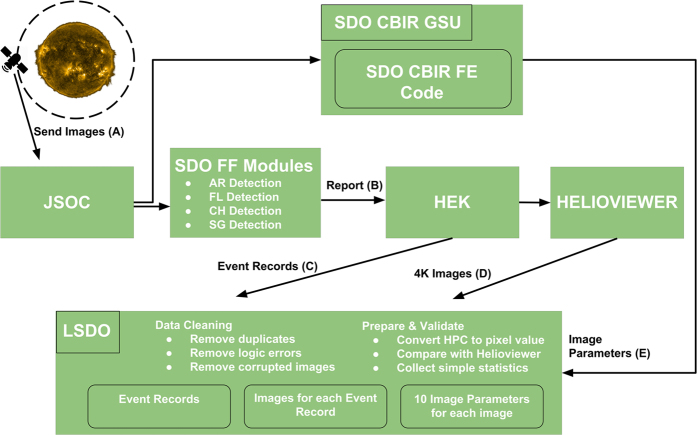
Complete process of data generation. (a) SDO satellite transfers full-disk image of the Sun to the Joint Science Operations Center (JSOC). (b) SDO Feature Finding modules reports detected events to HEK. (c) We extract event records from HEK. (d) We download three images for each record (a record may refer to same image). (e) We use the SDO CBIR GSU infrastructure to extract image parameters. SDO CBIR GSU is a content-based image retrieval module for solar image data hosted by Georgia State University, which extract image parameters from SDO images.

We start our dataset creation by choosing different event types which are reported to HEK. For each event type, we look for several different properties. First, the event should be detected by the FFT modules using SDO AIA images. Second, the event should occur frequently so that we collect a significant amount of images for each type. Third, the event reports should contain bounding box coordinates of the event so that computer vision applications can locate the event in the full-disk image. Using these criteria, we included four different event types: Active Region (AR), Coronal Hole (CH), Flare (FL), and Sigmoid (SG) to build our dataset. Figure 1 illustrates the complete process that was used to generate the dataset.

To collect event records of the four different event types, we used the public API provided by HEK (https://www.lmsal.com/hek/api.html). QueryHEK is a software package developed as client for the HEK API to retrieve the event records, which is open sourced in the following code repository (https://github.com/KarthikGP/QueryHEK). QueryHEK uses the HEK API and enables the specification of various constraints for any given query. We used event type and time constraints to retrieve all event records belonging to four chosen event types reported between January 1, 2012, and December 31, 2014.

**Figure 2:**
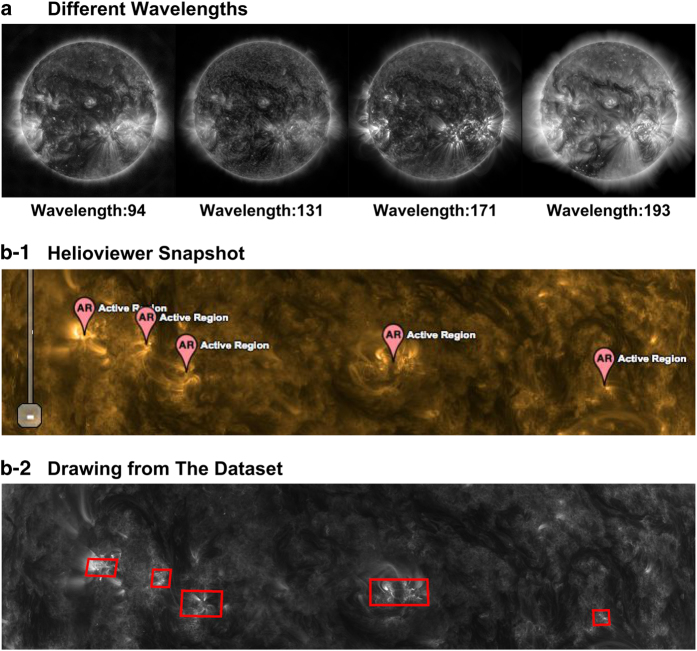
Solar image and event characteristics. (**a**) View of the Sun under different wavelengths at the same query time. (**b**) Solar event comparison between Helioviewer (**b-1**) and our dataset (**b-2**).

The SDO AIA instrument is designed to capture images of the Sun using eight different wavelength bands. These different wavelength bands reveal diverse characteristics of solar activities (see Table 1). The visual variability between four wavelength bands can be observed in Fig. 2a, showing the full-disk images taken during a small time window. The HEK data contains wavelength information for each reported event record, however, some records use composite wavelengths (e.g. ‘171 Å, 193 Å’). A certain small portion of the records does not have this information and has been discarded from this dataset. We investigated the most suitable wavelength for each event type according to the SDO FFT modules^2^. If an event record has multiple wavelengths or does not contain wavelength information, we assigned the wavelength of this record according to its event type.

The dataset choice of wavelengths is listed according to the selected event types in Table 1. In total, the dataset has 269,103 records. Event types by count can be seen in Table 1.

### High-resolution solar images

The main part of our dataset consists of high resolution images that are essential for computer vision applications. Helioviewer provides 4 K resolution images in JPEG 2000 format via their public API (http://helioviewer.org/api). We acquired images from Helioviewer’s API by specifying time and wavelength. If the exact time of the Helioviewer data does not overlap with the one that is specified, Helioviewer returns the image that is closest to the specified time. Helioviewer uses a reduced cadence to store images, preventing some certain event record times from fully overlapping with their data.

Each event record occurs in a certain time window indicated with a start and end time. However, the spatial location of an event record is unique. As the Sun rotates during this time window, the spatial location of the event changes. When the event start time does not overlap perfectly with the image available in Helioviewer, we are forced to select the image most closely available to the given event time. Instead of selecting a single image for each event record in the dataset, we include three images for each event record. Previous experimentation^7^ allowed us to present data that corresponds with a different stage of events. We believe that some types of research can benefit from this approach to create better learning models. Hence, we extract the three images for each event record at event start time, event end time, and the midpoint between start and end times. In total, we extracted 260 K JPEG 2000 images for 269 K event records. The image number discrepancy is due to the fact that sometimes multiple event records occur at the same time. We also kept the original Helioviewer image names in case users want to trace them back to their source.

### Image parameters

To be consistent with previous work in the solar image retrieval domain, we included ten different statistical image parameters for each image. These parameters have been used successfully on a wide variety of solar computer vision tasks as demonstrated in refs 6,8,9 and they have also been incorporated in previously released smaller datasets^7,10^. These parameters are calculated by dividing each image into 4,096 cells using a 64×64 grid. Ten parameters are extracted for each cell: Entropy, Mean, Standard Deviation, Fractal Dimension, Skewness, Kurtosis, uniformity, Relative Smoothness, Tamura Directionality, Tamura Contrast^6^.

### Code availability

In order for this dataset to be fully reproducible and expandable in the future, we have open-sourced all the Java code used to generate and validate the resource in the following code repository (https://github.com/ahmetkucuk/lsdo-generator).

The main code consists of three different components. The first component is the JPEG 2000 image downloader. This utility uses a list of desired events as input and downloads the corresponding images from Helioviewer. This code is multi-threaded for efficiency, which allows the user to specify the number of threads for downloading. The second component is the Image Drawer, which utility enables us to draw polygons of the event bounding boxes onto the corresponding image in JPEG or PNG format. This utility is used as part of our validation steps. The third component consists of several different independent routines used to clean and validate the dataset, such as the conversion of Helioprojective Coordinates (HPC) to pixel values, image de-duplication, etc.

In addition to our custom dataset generation code, we used QueryHEK, an open-source utility for retrieving events from HEK, and for the parameter extraction we used the tool developed in ref. 6. This code was originally written for extracting image parameters from NASA FITS files, but we converted the code to work with JPEG 2000 files. This new version of the tool is available in the following code repository (https://github.com/ahmetkucuk/parameter-extraction).

## Data Records

The dataset data records consist of three different parts: event records, corresponding images, and extracted image parameters. Event records encompass the list of solar events with generated and extracted attributes. We included the generated attributes to make it easier to work with the resource. The extracted attributes are transferred directly from the available HEK meta-data. Table 2 presents these attributes. Among these attributes, *KB Archive ID* is an unique ID of the event used by HEK. *Start Time* and *End Time* are the beginning and end times of the events. Another attribute is *Wavelength*, which allows us to identify the AIA wavelength of the image that is appropriate for the record. *Reported From* indicates the FFT module that reports the corresponding event to HEK. This attribute is very useful as different modules are detecting and reporting the same events at times, allowing users to distinguish the duplicate reports from the events easily.

The bounding box attribute consists of four coordinate pixel values. We have pre-processed all original polygon values of event records from their source HPC solar coordinate system into pixels. This pre-processing step is vital to make polygons compatible with the high-resolution images in the dataset. We used Java code for this conversion that was adapted from the SunPy library (http://sunpy.org/). A Bounding Box is provided as textual representation of the geometry according to Open Geospatial Consortium (OGC) standards.

For easy access to the image and image parameters files, we specify the file name of start time, middle time, and end time image files for each event record so that users can find the image of the event record without needing the temporal search among the images. Image files are separated into the different directories for easy access and maintenance. First, we separate images into wavelengths. Then, we separate them according to the day of the event. For example, images of AR event record in wavelength 171 Å that occur on 4 May 2013 are stored in *lsdo/images/SDO/AIA/171/2013/05/04/*directory. The image parameters have a consistent directory structure, for example, image parameters of the AR event are stored under the *lsdo/parameters/SDO/AIA/171/2013/05/04/*.

For distribution of the dataset, the folder structure consists of three main folders: images, events, parameters. All meta-data is provided as tab-delimited files and all images are provided in JPEG 2000 format. In Table 3, we list ten separate files that are stored on Harvard Dataverse (Data Citations 1–10). Alternatively all zipped dataset files and the source code is publicly available and can be downloaded from the project website (http://lsdo.dmlab.cs.gsu.edu/). Total size of the dataset is 284 Gigabytes, making it the largest solar data resource publicly available in one place.

## Technical Validation

We conducted several cleaning and validation processes to have a reliable dataset. Our methodology follows the ones developed for prior datasets, which only provide image parameters and not full-disk images of the Sun^7,10^. The methodology has been adapted and is described in the following paragraphs.

When extracting relevant solar events, the raw data retrieved from HEK is not free from errors. The first problem is that HEK contains duplicate events with the same start time, end time, and the same polygon values. We removed these duplicate events from the data. Some of the event records have suspiciously longer duration considering the nature of the solar phenomena and the design of FFT detection modules. To avoid adding noise to our dataset, we considered these records as false by filtering and removing events with duration longer than six days. A similar issue was also examined in ref. 7. As with any real-world data, we also encountered events that have an end time before their start time, which is logically incorrect and was therefore eliminated as well.

In terms of the image data, while Helioviewer API serves image data generated by the AIA module with high temporal resolution, this repository does not guarantee to provide the exact image taken at the requested time. Instead, Helioviewer provides the closest image to the requested time, due to the time cadence Helioviewer uses for the images it stores. In addition to the images, Helioviewer API provides meta-data which consists of the date of the image, the module taken from, and the wavelength. When comparing the date of the downloaded image with the actual date of the event, we observed that there are some events that have long gaps between these two dates. If there is a long gap between the date of an event and the corresponding image, projecting the event bounding box on the image does not give us the precise location, because of the Sun’s rotation. Having these kinds of problematic data might cause serious deficiencies on the event bounding boxes provided. To avoid this, we removed the events that have a gap larger than 10 min for start time, mid time, or end time. This removed around 10 K events while making the dataset much cleaner and more reliable. When downloading massive amounts of images using Helioviewer’s API, there are some issues with denied requests that lead to data corruption. To address this issue, we repeated the download procedure for the corrupted images. This was automated as the relative image file size of a corrupted image is considerably smaller than the average image file size. However, after multiple attempts, we discovered that around 400 are not properly stored in Helioviewer, so we removed the respective events in order to avoid corrupted image files.

In order to validate the bounding box attribute of event records, we checked the validity of polygons according to the OGC specification using the Java Topology Suite (JTS) library^11^. We found that around 7 K polygons were not valid according to the JTS library. Our investigation showed that these polygons were representing a line instead of a surface, which might be a correct module output (an event very close to the limb), but it is not helpful for computer vision tasks. We removed these records from the dataset. Another step to test the validity of polygons was to overlay them visually onto the corresponding image. We randomly selected samples of events from each event type and wavelength, and we drew polygons onto the images. We visually compared our drawing results with the Helioviewer web application. Figure 2b shows one of our test cases where a region of the Sun can be observed based on both Helioviewer records and our data. With this visual validation, we are confident about the conversion between HPC to pixel values because of the right position of the bounding boxes.

In order to test the image parameter extraction, we evaluated the software developed in ref. 6 against our adapted version of this software that works on JPEG 2000 files. Since there are minor differences on how the original NASA FITS file software normalizes the images versus how Helioviewer normalizes the images, we get minor variations on the parameter values, but the overall impact is negligible as it has been demonstrated in ref. 12.

**Figure 3:**
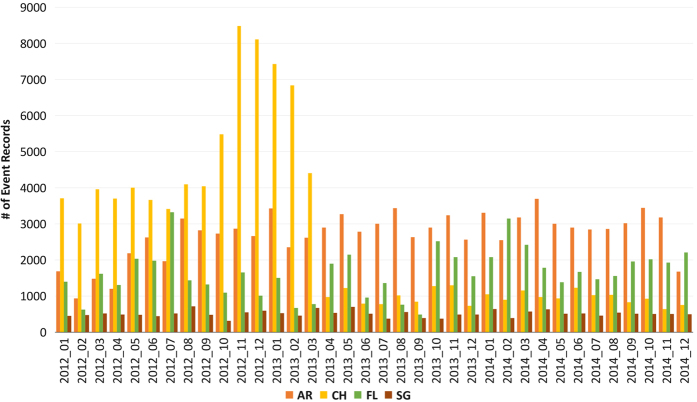
Number of the events by month and event type.

In Fig. 3 we present event counts to observe the distribution of our event records by time. We can observe that the dataset contains a mostly even distribution of event records for each month. In addition, Table 4 shows simple statistics on duration and area of regions that are collected for each event type in the dataset. The increase of Coronal Hole (CH) events during the last few months of 2012 and the beginning of 2013 shows an interesting pattern that does not seem to repeat, however, the event reports have been double checked and are properly attributed.

## Usage Notes

By providing a large repository of curated solar events, images, and statistical image parameters, our resource aims to facility large-scale solar research. In the past we have released smaller datasets, which now are a small part of this resource, but we never included full-resolution images. With this addition we expect the computer vision community to be able to take advantage of this large-scale repository as the first one in the solar physics domain. In the next sections we showcase different ways in which such a dataset has been used at a smaller scale.

### Large-scale solar image retrieval

In order to build an efficient and scalable Solar CBIR system with the purpose of allowing researchers to search for similar images within the SDO image repository (currently with over 100 million images), we needed a considerable amount of labeled image data^13^. The CBIR system was built using a smaller dataset, and could have greatly benefited from using a larger resource such as this one. Our initial step when building the system used labeled image date to identify which image parameters are the most efficient, in terms of cost and retrieval accuracy, for such a task^8^. Having extensively validated our image parameter selection, this resource provides also provides them for scientists who are more interested in retrieval than image processing. With newer and more complex computer vision and image processing algorithms developed each year, we are addressing the need for truly large scale datasets that provide multi-modal data for scientists to evaluate their algorithms. The availability of Large Scale Solar Dynamics Observatory Image Dataset for computer vision applications (LSDO) will greatly help researchers in the solar physics domain, as there has never been such an extensive resource available for download or solar images and solar event labels combined.

### Finding regions of interest in solar images

As another active area in the computer vision and image retrieval fields, finding Regions of Interest (ROI) is another perfect example of the need of having a large labeled training corpus. In our previous work^9^ and^8^, we have investigated how to use unsupervised learning techniques for finding regions of interest within the solar images that contain potential solar phenomena. By experimenting with multiple methods, we have developed a successful approach to automatically detecting ROIs for a more refined and robust search in the CBIR system. Research work like this would not be possible without the availability of labeled datasets.

### Comparing image parameters between solar and medical images

A small subset of the presented dataset has been used in the past^14^ to demonstrate the transferability of image parameters used within multiple domains for retrieval purposes. This work demonstrated that the included image parameters could be extracted on medical radiographs and used to separate those images in multiple accurate classes with nearly 85% classification accuracy. We provide the code to extract such image parameters from any type of images with this resource. We theorize that other parameters found to be effective in other domains can benefit Solar Physics and vice versa, our resource facilitates these types of analyses by providing several hundred thousand labeled events and images.

### Flexibility of the resource

By providing full size images, we allow researchers to fine-tune object recognition and other computer vision algorithms without the constraints of only having the regions of interest. Using high-resolution (4 K×4 K) images, researchers are able to down-sample the images freely and are able to test algorithms with a wide range of settings and parameters. By providing multiple wavelengths and the raw image parameters used in the SDO CBIR system^15^, we provide multiple levels of data that could facilitate research in both traditional image retrieval, similarity evaluation, and on more modern topics, such as Convolutional Neural Networks and other Deep Learning applications.

Finally, another major advantage of our resource is that we provide all source code for researchers to periodically add new annotated SDO data. By releasing all documentation and code, we are enabling all researchers to update the dataset when needed, rather than having them wait for our group to release a new version (when funding and time permits).

## Additional Information

**How to cite this article**: Kucuk, A. *et al.* A large-scale solar dynamics observatory image dataset for computer vision applications. *Sci. Data* 4:170096 doi: 10.1038/sdata.2017.96 (2017).

**Publisher**’**s note**: Springer Nature remains neutral with regard to jurisdictional claims in published maps and institutional affiliations.

## Supplementary Material



## Figures and Tables

**Table 1:** Wavelength and record count per event type.

**Event Type**	**Wavelength**	**Number of the Events**
Active Region	171 Å	97,022
Coronal Hole	193 Å	94,693
Flare	193 Å	59,069
Sigmoid	94 Å	18,319
Total		269,103

**Table 2:** List of attributes in each event record.

Extracted Attributes	KB Archive ID
	Event Type
	Start Time
	End Time
	Wavelength (in Å)
	Bounding Box
	Reported From
Derived Attributes	Start Time Image
	Middle Time Image
	End Time Image

**Table 3:** List of LSDO datasets in Harvard Dataverse.

**Dataset Title**	**Description**	**DOI**
LSDO Sample	3 days sample from LSDO	http://dx.doi.org/10.7910/DVN/8XRUDT
LSDO Event Records	Event records for: AR, CH, FL, SG	http://dx.doi.org/10.7910/DVN/BDRJRZ
LSDO images at wavelength 94 Å	Full disk AIA images at wavelength 94 Å	http://dx.doi.org/10.7910/DVN/OROOPU
LSDO images at wavelength 131 Å	Full disk AIA images at wavelength 131 Å	http://dx.doi.org/10.7910/DVN/TECTSL
LSDO images at wavelength 171 Å	Full disk AIA images at wavelength 171 Å	http://dx.doi.org/10.7910/DVN/FVEFXI
LSDO images at wavelength 193 Å	Full disk AIA images at wavelength 193 Å	http://dx.doi.org/10.7910/DVN/HYJTFW
LSDO images at wavelength 211 Å	Full disk AIA images at wavelength 211 Å	http://dx.doi.org/10.7910/DVN/AVA3F3
LSDO images at wavelength 304 Å	Full disk AIA images at wavelength 304 Å	http://dx.doi.org/10.7910/DVN/LGKQSG
LSDO images at wavelength 335 Å	Full disk AIA images at wavelength 335 Å	http://dx.doi.org/10.7910/DVN/ZL9ZRV
LSDO Image Parameters	10 image parameters extracted from all full-disk AIA images	http://dx.doi.org/10.7910/DVN/ANCVVY

**Table 4:** Statistics obtained from bounding box attribute and duration of the event records.

**Type**	**Area (pixel squared)**				**Duration (hours)**			
	**Min**	**Mean**	**Max**	**s.d.**	**Min**	**Mean**	**Max**	**s.d.**
AR	0.001	55.892	1478.530	97.785	0.200	3.978	5.977	0.401
CH	0.001	97.415	3838.428	225.051	0.000	1.482	5.883	1.938
FL	0.012	25.557	4086.462	60.447	0.033	0.225	5.973	0.418
SG	7.656	39.348	1817.103	34.493	0.000	0.500	4.233	0.031
